# Enhanced performance of sulfur-infiltrated bimodal mesoporous carbon foam by chemical solution deposition as cathode materials for lithium sulfur batteries

**DOI:** 10.1038/srep42238

**Published:** 2017-02-06

**Authors:** Tae-Gyung Jeong, Jinyong Chun, Byung-Won Cho, Jinwoo Lee, Yong-Tae Kim

**Affiliations:** 1 School of Mechanical Engineering, Pusan National University, Busan 609-735, Republic of Korea; 2 Center for Energy Convergence, Korea Institute of Science and Technology (KIST), Seoul 02792, Republic of Korea; 3 Department of Chemical Engineering, Pohang University of Science and Technology (POSTECH), Pohang 37673, Republic of Korea

## Abstract

The porous carbon matrix is widely recognized to be a promising sulfur reservoir to improve the cycle life by suppressing the polysulfide dissolution in lithium sulfur batteries (LSB). Herein, we synthesized mesocellular carbon foam (MSUF-C) with bimodal mesopore (4 and 30 nm) and large pore volume (1.72 cm^2^/g) using MSUF silica as a template and employed it as both the sulfur reservoir and the conductive agent in the sulfur cathode. Sulfur was uniformly infiltrated into MSUF-C pores by a chemical solution deposition method (MSUF-C/S CSD) and the amount of sulfur loading was achieved as high as 73% thanks to the large pore volume with the CSD approach. MSUF-C/S CSD showed a high capacity (889 mAh/g after 100 cycles at 0.2 C), an improved rate capability (879 mAh/g at 1C and 420 mAh/g at 2C), and a good capacity retention with a fade rate of 0.16% per cycle over 100 cycles.

Lithium sulfur batteries (LSB) have received attention as next generation energy storage device because of higher capacity (1675 mAh/g) and energy density (2600 Wh/kg) than conventional Li-ion battery^1^,^2^. However, the main drawback of LSB is poor cyclability because the lithium polysulfide is easily dissolved in the electrolyte during charge-discharge process^3^,^4^,^5^,^6^.

Most of researches have focused on how to prevent the polysulfide dissolution and to enhance the cyclability. To prevent the polysulfide dissolution, various approaches have been suggested, such as employment of mesoporous carbon, carbon coating and polymer coating. Among these, an employment of mesoporous carbon has successfully suppressed the polysulfide dissolution by spatial confinement of sulfur in the electrochemically accessible mesopores^7^,^8^,^9^,^10^,^11^,^12^,^13^. Since Nazar *et al*. introduced a mesoporous carbon host (CMK-3) for sulfur encapsulation^7^, this approach has demonstrated a great improvement in cyclability by preventing the polysulfide dissolution. To date, various types of carbon matrix have been examined as sulfur reservoirs for the composite cathode of LSB. However, most of reported micro-mesoporous carbon/S composites had very low sulfur content because of small pore volume, which is not suitable for achieving a high energy density^14^,^15^. Li *et al*. prepared the peapodlike mesoporous carbon with large pore volume and sulfur was infiltrated into pores by melt-diffusion method for preparation of S/C composite. Although the composite was included more than 70 wt% sulfur, it had the problem that the capacity is continuously decreased by polysulfide dissolution. That is, a large pore size is advantageous for the sulfur loading amount but disadvantageous for the polysulfide confinement. On the contrary, a small pore size has a directly opposite feature to the case of large pore size. Hence, it is desirable for the sulfur reservoir to have a proper combination of characteristics of both large and small pore size.

In this study, we examined a new type of mesoporous carbon foam as the sulfur reservoir having bimodal mesopores (4 and 30 nm), synthesized with mesocelluer silica foam (MSUF) as a template, referred to as MSUF-C. The surface nature of MSUF-C was changed from hydrophobic to hydrophilic thorough an acid treatment in order to form stronger bonding between carbon and sodium thiosulfate pentahydrate (sulfur precursor). Sulfur was infiltrated into the pores in MSUF-C with three methods; a ball mixing (BM), a melt diffusion (MD), and a chemical solution deposition (CSD), referred to as MSUF-C/S BM, MSUF-C/S MD, and MSUF-C/S CSD, respectively.

Among the prepared samples, the MSUF-C CSD particularly demonstrated a well-balanced feature of large pore size being beneficial for sulfur loading and small pore size having a merit of polysulfide confinement. The CSD approach with a sodium polysulfide precursor solution led to a uniform infiltration of sulfur even into 4 nm pore as confirmed by Small-angle X-ray scattering (SAXS) and therefore a marked increase of sulfur loading up to 73 wt.%. On the other hand, BM or MD methods could not fully infiltrate the sulfur into the 4 nm pore. As a result, the MSUF-C/S CSD exhibited enhanced cyclability and retained a stable capacity of 889 mAh/g after 100 cycles.

## Results and Discussion

### Structural analysis of MSUF-C and MSUF-C/S composites

**Figure 1:**
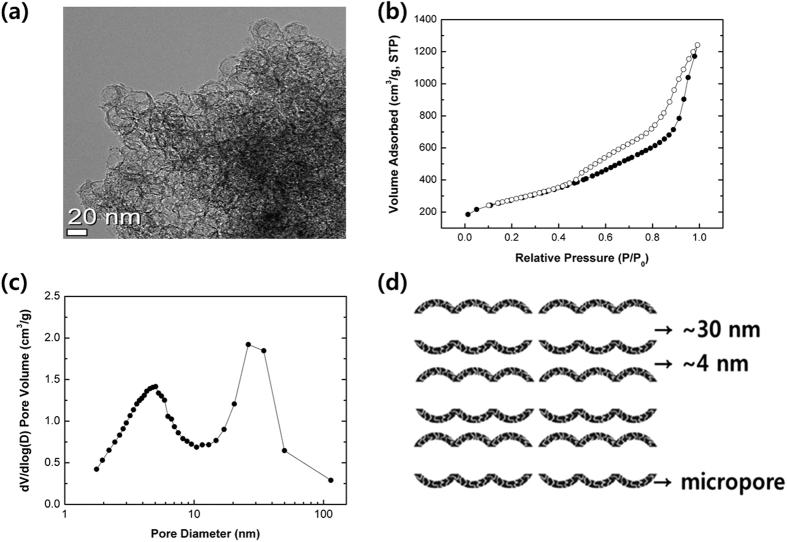
(**a**) TEM image of MSUF-C. (**b**) Nitrogen adsorption-desorption isotherm and (**c**) pore size distribution obtained using BJH method from the adsorption branch of the N_2_ adsorption isotherm. (**d**) Schematic diagram of MSUF-C structure.

Transmission electron microscopy (TEM) and nitrogen adsorption measurement was used to investigate the morphology and particle size of synthesized MSUF-C, as shown in Fig. 1. In Fig. 1(a), TEM images show that the particle size of MSUF-C is around 30 nm and MSUF-C has the interconnected mesoporous channels. This mesoporous carbon with interconnected nanochannels has been reported conducting networks for transference of Li ion and electron^16^. In addition, the flexibility of the mesoporous carbon sufficiently alleviates the structure degradation caused by the volume expansion of sulfur.

Pore structure of MSUF-C was characterized with nitrogen adsorption studies. Figure 1(b) and (c) show the nitrogen adsorption isotherm and pore size distribution of MSUF-C calculated using the BJH (barrett-Joyner-Halenda) method. The nitrogen adsorption/desorption isotherms of MSUF-C (Fig. 1(b)) exhibit hysteresis at P/P_0_ = 0.7 and P/P_0_ = 0.9. The hysteresis loop at P/P_0_ = 0.7 and P/P_0_ = 0.9 are contributed to the small mesopores caused by dissolution of the microcellular silica walls and the large mesopore, respectively^17^. The pore size distribution (Fig. 1(c)) calculated from the nitrogen isotherms using the BJH method. Pore size distribution plot exhibited two narrow peaks centered at 4 nm and 30 nm, which clearly indicates that both the small and large pore were successfully formed. The small pore (4 nm) can be expected to efficiently inhibit the polysulfide dissolution. In addition, the large pore (30 nm) can provide a sufficient space to accommodate the active material, leading to high loading density. The schematic diagram of MSUF-C structure was presented in Fig. 1(d), based on the TEM and the pore size distribution results, showing that MSUF-C consists of bimodal structure with 4 nm small pore and 30 nm large pore.

In order to prepare the MSUF-C/sulfur composites, sulfur was infiltrated into pore of MSUF-C via various methods, such as a simple ball mixing (MSUF-C/S BM), a melt diffusion method (MSUF-C/S MD)^18^ and a chemical solution deposition method (MSUF-C/S CSD). The prepared MSUF-C/S composites were observed by TEM as shown in Figure S1. The morphology of MSUF-C in MSUF-C/S BM or MSUF-C/S MD was slightly defecting owing to the harsh preparation condition and deformity. On the other hand, the spherical morphology of MSUF-C in MSUF-C/S CSD was well retained without defect. This indicates that a synthetic condition of CSD-method is enough mild not to affect the morphology of MSUF-C, compared to other synthesis methods. The unchanged structure of MSUF-C could be beneficial for the transfer of lithium ion and electron in cathode. In addition, sulfur was well encapsulated into MSUF-C pores and therefore polysulfide may be less soluble into electrolyte.

**Figure 2:**
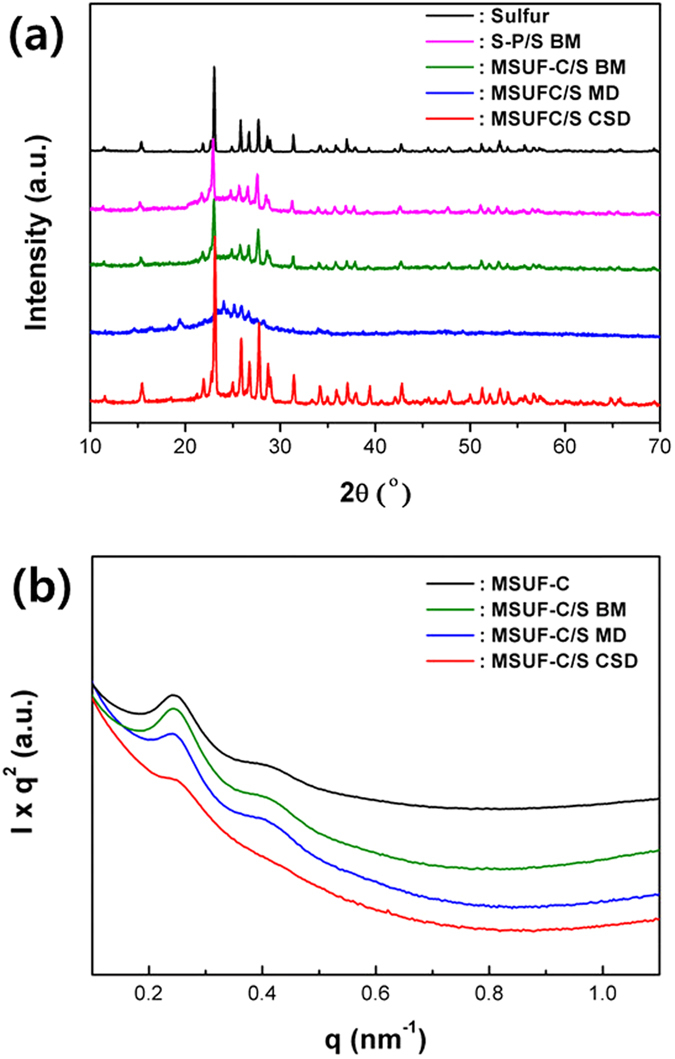
(**a**) X-ray diffraction (XRD) and (**b**) small angle x-ray scattering (SAXS) patterns of different C/S composites.

The difference of crystal structure of MSUF-C/S composites was observed by X-ray diffraction (XRD) and Small angle x-ray scattering (SAXS) patterns in Fig. 2. Additionally, Super-P/S simple ball-mixing composite (S-P/S BM) as pristine electrode was synthesized for the comparison. Super-P carbon was mixed with sulfur by only simple ball-mixing, because the carbon has no characterization of porous structure. As shown in Fig. 2(a), elemental sulfur generally exhibits in a crystalline state with an Fddd orthorhombic structure^19^. The sulfur in S-P/S BM, MSUF-C/S BM and MSUF-C/S CSD show a diffraction pattern corresponding to the orthorhombic structure, indicating that the process for the C/S composite did not occur any structure transformation for sulfur. The diffraction patterns for all the prepared samples were similar each other, but the peak intensity of sulfur in MSUF-C/S CSD is markedly higher than that of S-P/S BM and MSUF-C/S BM. This implies that the CSD approach is more beneficial to form a homogenous sulfur crystal than other methods. On the other hand, the diffraction peak of sulfur in MSUF-C/S MD disappeared and the broad peak around 2θ = 24° was observed, suggesting that the sulfur exists in an amorphous state after melting^20^,^21^.

The SAXS patterns of MSUF-C show two diffraction peaks at 0.25 nm and 0.4 nm, as shown in Fig. 2(b). The diffraction peak at 0.25 nm and 0.4 nm is corresponding to the large pore (30 nm) and the small pore (4 nm), respecively^22^,^23^. The SAXS pattern of MSUF-C/S BM was not changed, indicating that the sulfur is only coated on surface of MSUF-C, not in pore. That of MSUF-C/S MD at 0.25 nm is slightly decreased, suggesting that some sulfur is infiltrated into only the large pore (30 nm) of MSUF-C^7^,^24^. It is however interesting to note here that no SAXS pattern was detected for the MSUF-C/S CSD, demonstrating that all the pores of MSUF-C were perfectly filled with sulfur. Based on SAXS results, the schematic diagrams of different MSUF-C/S composites were presented in Figure S2.

### Electrochemical performances of C/S composites

To examine the sulfur content in MSUF-C/S composites, TGA analysis was carried out. As shown in Figure S3, the weight loss of MSUF-C/S MD and MSUF-C/S CSD were 65.46% and 72.86%, respectively, indicating that the CSD approach is more effective for the sulfur infiltration than other methods.

**Figure 3:**
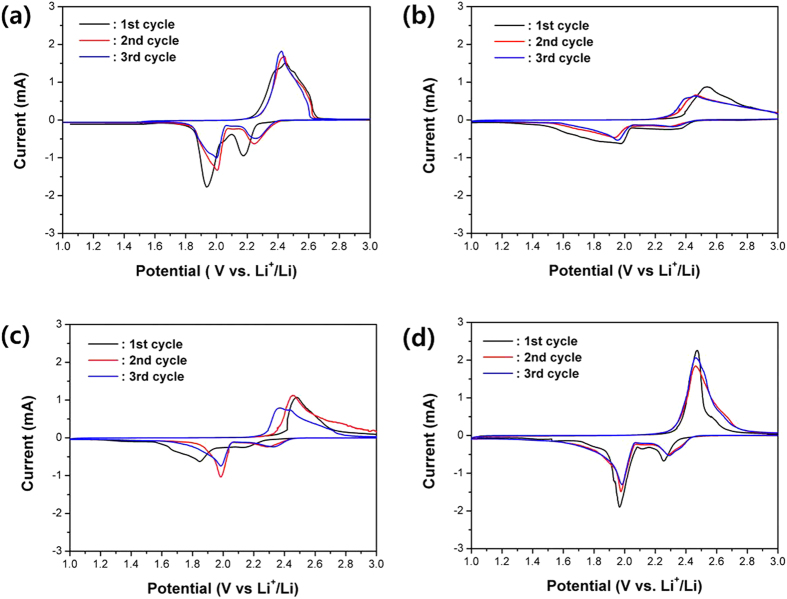
Cyclic voltammogram of (**a**) S-P/S MD, (**b**) MSUF-C/S BM, (**c**) MSUF-C/S MD, and (**d**) MSUF-C/S CSD cells at scan rate of 0.1 mV/s.

To evaluate the electrochemical properties of prepared samples, we performed electrochemical measurements using coin-type cell. The cyclic voltammogram (CV) curves of S-P/S BM and MSUF-C/S composites range of 1 from 3 V at a scan rate of 0.1 mV/s exhibits two main cathodic peaks at 2.4 V and 2.0 V as shown in Fig. 3, which are attributed to the transformation of elemental sulfur to long chain lithium polysulfides (Li_2_Sn, n > 4) and reduction of lithium polysulfides to insoluble lithium sulfide (Li_2_S_2_, Li_2_S), respectively^18^,^21^,^25^,^26^. It is noteworthy that MSUF-C/S CSD showed much less change of CV curves with cycles than other samples, indicating that the sulfurs were infiltrated into the pores homogeneously. In the case of MSUF-C/S CSD, peaks are observed at 2.3 V and 2.0 V during cathodic scan of the first cycle. During the reverse scan, sharp peak is observed at 2.6 V. In comparison with the other samples, the redox peak shape was much sharper, implying that the kinetics of lithium sulfur formation is much faster than other samples. This higher kinetics can be also supported the fact that the potential of MSUF-C/S CSD was higher for cathodic peaks (discharge process) and lower for anodic peak (charge process) than other samples. The peak intensity of 2^nd^ and 3^rd^ cycles was maintained during the redox reaction, indicating that MSUF-C/S CSD has a markedly higher utilization of sulfur and reversible electrochemical sulfur reaction. These results evidently demonstrate that MSUF-C/S CSD has a good electrical contact between sulfur and carbon and then inhibited the dissolution of polysulfide.

**Figure 4:**
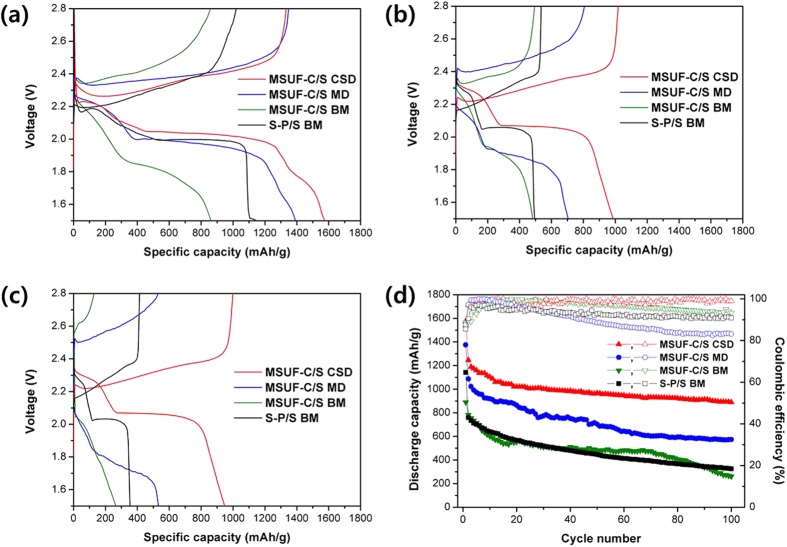
Electrochemical performances of S-P/S BM and MSUF-C/S composite cells. (**a**) 1st, (**b**) 50th, (**c**) 100th discharge/charge profiles and (**d**) cycling performances at 0.2 C rates.

Battery performance of prepared samples was evaluated using conventional galvanostatic test at 0.2C. Figure 4 represents the voltage profiles and cycle performance of lithium sulfur cell during the charge/discharge process. Two distinct plateaus are observed in the discharge curves of the cells, which are well consistent with the CV results. The discharge capacities for the first cycle and after 100 cycles were 1142 mAh/g and 325 mAh/g (57.4% of capacity retention on the basis of 20^th^ cycle) for S-P/S BM, 889 mAh/g at first discharge and 261 mAh/g (47.2% of capacity retention on the basis of 20^th^ cycle) for MSUF-C/S BM, 1375 mAh/g at first discharge and 572 mAh/g (66.2% of capacity retention on the basis of 20^th^ cycle) for MSUF-C/S MD, and 1575 mAh/g at first discharge, 889 mAh/g (87.1% of capacity retention on the basis of 20^th^ cycle) for MSUF-C/S CSD, respectively. As can be seen in these data set, MSUF-C/S CSD exhibited much better discharge capacity and capacity retention than other samples. MSUF-C/S CSD achieved the high loading density of active sulfur material and simultaneously was successfully inhibited the polysulfide dissolution in comparison with other samples, because sulfur was uniformly infiltrated into small pores of MSUF-C with no defect and no deformation by chemical solution deposition method, as can be seen in TEM and SAXS result. On the other hand, the sulfur particles in the carbon/sulfur composites synthesized by simple ball mixing exist on the surface of MSUF-C due to no infiltration into the carbon. Moreover, sulfur is difficult to embed into Super-P carbon with no porous structure. The sulfur particles nonembedded in the carbon therefore can be easily diffused out the electrode and deposited on Li metal anode during cycling, resulting in poor capacity retention.

### Electrochemical Impedance Techniques

It is widely recognized that the cycle stability of lithium ion batteries is attributed to the interfacial charge transfer and lithium ion diffusion^27^. Electrochemical impedance spectroscopy (EIS) studies were carried out in order to check the interfacial resistance of MSUF-C/S composites. The impedance spectra were recorded at the open circuit potential before and after cycling. (see Figure S4) The EIS spectrum consists of one semicircle at high frequency region corresponding to the charge transfer resistance (resistance of the electrode surface) and short inclined line at low frequency region to the ion diffusion in electrode^28^. The cell for MSUF-C/S CSD shows lower interfacial resistance than other cells, indicating again that the good electrical contact between sulfur and carbon facilitates faster charge transfer through the electrode/electrolyte interface. With the increase of cycle number, the difference in charge transfer resistance became gradually broader between MSUF-C/S CSD and other samples. The highest sulfur trapping ability of MSUF-C/S CSD can be also confirmed with the change of electrolyte bulk resistance corresponding to the first Z’-intercept in the x-axis. The initial bulk resistance was identical for all the samples, while a clear difference was shown with the increase of cycle number. After 100 cycles, the electrolyte resistance determined by extrapolation of the Nyquist plot to the initial frequency had 21.20 ohm (MSUF-C/S BM), 13.02 ohm (MSUF-C/S MD) and 5.75 ohm (MSUF-C/S CSD), respectively. The lower bulk resistance for MSUF-C/S CSD than other samples is attributed to the fact that the polysulfide is less soluble into the electrolyte during cycling. Therefore, it can be confirmed again that MSUF-C/S CSD could inhibit polysulfide dissolution into electrolyte.

### Rate capability of MSUF-C/S composites

**Figure 5:**
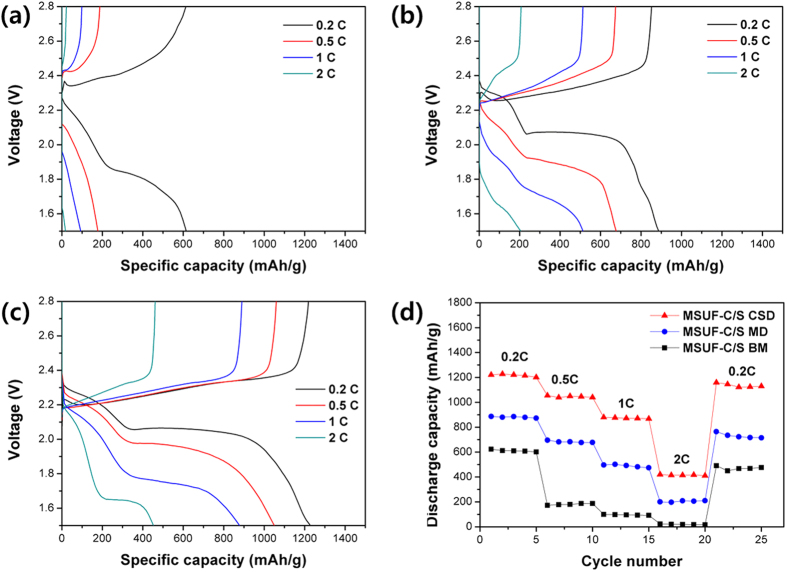
Charge and discharge curves of (**a**) MSUF-C/S BM, (**b**) MSUF-C/S MD and (**c**) MSUF-C/S CSD cell. (**d**) Rate capacility of MSUF-C/S composite cells at various current rates from 0.2 C to 2 C.

Finally, the rate performances of the MSUF-C/S composites were examined at various current densities, as can be seen in Fig. 5. Discharge capacities at 2C were 23.01 mAh/g (3.83% of that at 0.2C) for MSUF-C/S BM, 208 mAh/g (23.1%) for MSUF-C/S MD, and 420 mAh/g (35%) for MSUF-C/S CSD. The MSUF-C/S CSD demonstrated a much enahanced rate capability than other samples, which is attributed to lower charge transfer resistance due to good electrical contact between sulfur and mesoporous carbon.

In summary, we synthesized the bimodal mesoporus carbon foam (MSUF-C) with large pore (30 nm) and small pore (4 nm). Sulfur was infiltrated into synthesized mesoporous carbon using various methods, such as the simple ball-mixing, the melt-diffusion and the chemical solution deposition method. In comparison with the simple ball-mixing and the melt diffusion method, the MSUF-C synthesized by CSD-method had less surface defect and structural distortion, and the sulfur was uniformly dispersed in mesopores of MSUF-C, as confirmed by TEM and SAXS analysis. The sulfur with MSUF-C/S CSD loaded in mesopores as high as 73% on the basis of TGA result, due to the large pore volume and uniformly dispersed sulfur by CSD-method. MSUF-C/S CSD resulted in higher capacity, capacity retention and rate capability than other samples and showed enhanced cycle perforamnce by suppressing the polysulfide dissolution. This result can be attributed to the fact that the sulfur was uniformly infiltrated into even small pores of MSUF-C by CSD-method. Also, MSUF-C/S CSD showed enhanced rate capability because of lower charge transfer resistance from good electrical contact between sulfur and mesoporous carbon. MSUF-C/S CSD achieved high loading density of sulfur by large pore volume and CSD approach, and successfully prohibited the polysulfide dissolution thanks to the sulfur imbedded into small pores of MSUF-C. Hence, a combination of the MSUF-C with bimodal mesopores and the CSD-method can be a promising solution to achieve a long cycle life and a high rate capability for lithium sulfur batteries.

## Methods

### Synthesis of MSUF-C

MSUF-C was synthesized following the procedure reported by Kim *et al*.^29^,^30^,^31^. After calcination at 550 °C for 4 h, alumination (Si/Al = 20) was performed, by means of the impregnation method, to generate acidic catalytic sites for the polymerization of furfuryl alcohol inside the mesopores. 1 g of aluminated MSU-F silica is wetted with 2 ml furfuryl alcohol using the incipient wetness technique, and is then polymerized at 80 °C for 12 h. The resulting aluminated MSU-F composite was carbonized at 850 °C for 2 h under a nitrogen atmosphere and etched by HF solution (5 wt%) to generate MSUF-C.

### Acid treatment

Acid treatment was carried out using nitric acid. Acid solution was prepared at 3.0 M. For the acid treatment, 0.5 g of MSUFC was mixed with 100 M*l* of the acid solution by mechanically stirring for 6 h. Then, the solution was filtered and washed with DI water. The filtered MSUF-C was dried in oven at 80 °C.

### Synthesis of MSUF-C/Sulfur composite

#### Chemical solution deposition method (CSD-method)

Mesoporous carbon/sulfur composite was prepared by a chemical solution deposition method in an aqueous solution: Na_2_S_2_O_3_(aq) +2HCl −>S(s) +SO_2_(g) +2NaCl(aq).

Sodium thiosulfate penta hydrate (Aldrich, 10 g) was dissolved in DI water (100 ml) and then the as-prepared MSUF-C (0.5 g) was added. The mixture was homogeneously dispersed using a magnetic stirrer for 1 h. Well dispersed mixture was filtered and washed with DI water several times and dried at 80 °C for 12 h in a vacuum oven. Dried powder and cetyltrimethylammoniumbromide (CTAB) were added in HCl solution (2.48 ml of HCl in 100 ml of water) and was stirred. The precipitate was filtered and washed with DI water to eliminate salts and impurities, and was dried at 80 °C in a vacuum oven.

#### Melt-diffusion method

Mesoporous carbon (MSUF-C, 0.3 g) and sulfur (Aldrich, 0.9 g) were homogeneously mixed by ball mixer. Mixed carbon/sulfur powder was thermally treated at 155 °C for 2 h.

#### Simple Ball-mixing

Sulfur (Aldrich, 0.9 g) was mixed with mesoporous Carbon (MSUF-C, 0.3 g) or Super-P carbon (0.3 g) using a ball mill-mixer (pulverisette 23).

### Characterization

TEM image were taken using Tecnai F20 G^2^ (accelerating voltage 200 kV), respectively. Crystalline structure of the samples was characterized by X-ray Diffraction (Rigaku D MAX-2500/PC, 40 kV, 100 mA Cu-ka). Small angle X-ray scattering (SAXS) was collected using a PANalytical PW3830 (40 kV, 30 mA Cu-ka). Nitrogen adsorption-desorption isotherms and pore size distributions were measured at 77 K using a Micromeritics ASAP2000 analyzer and calculated using BJH (Bareet-Joyner-Halenda) method from N_2_ adsorption branches. Thermal gravimetric analysis (TGA) was conducted on a TA instruments.

### Electrochemical measurements

To prepare the cathode for lithium sulfur battery, 80 wt.% of MSUF-C/S composites were mixed with 10 wt.% of conductor (Super P) and 10 wt.% of binder (poly vinyliden fluoride) using a ball-mixer (pulverisette 23) in N-methyl-2pyrrolidinone to form a slurry. The slurry was coated on aluminum foil using a doctor blade and dried in a vacuum oven at 60 for 12 h. The loading density of sulfur was about 1 mg/cm^2^. 2032-type coin cells were assembled in a drying room using lithium foil on copper as the counter electrode. Polyethylene membrane used as the separator was obtained from SK-innovation Inc. The electrolyte used was 1 M Lithium bis(trifluoromethan sulfonyl) imide in tetra (ethylene glycol) dimethyl ether and 1,3-dioxolane (1:1 v/v) containing 0.2 M LiNO_3_. The electrolyte volume of 60 μ*l* was injected into the 2032 coin cell. Galvanostatic cycling was carried out using a battery tester (Maccor 4300 K) from 1.5–2.8 V versus Li^+^/Li at 0.1 C (1 C = 1675 mAh/g). Cyclic voltammetry (CV) measurements were performed on solartron 1286 at a scan rate of 0.1 mV/s. Electrochemical Impedance Spectroscopy (EIS) measurement were performed using Solartron 1260 impedance gain-phase analyzer in combination with Solartron 1286 within the frequency range of 1 MHz to 100 mHz at amplitudes of 5 mV.

## Additional Information

**How to cite this article**: Jeong, T.-G. *et al*. Enhanced performance of sulfur-infiltrated bimodal mesoporous carbon foam by chemical solution deposition as cathode materials for lithium sulfur batteries. *Sci. Rep.* **7**, 42238; doi: 10.1038/srep42238 (2017).

**Publisher's note:** Springer Nature remains neutral with regard to jurisdictional claims in published maps and institutional affiliations.
